# Multi-perspective views about healthcare experiences for those with incurable head and neck cancer: A prospective, longitudinal, qualitative study

**DOI:** 10.1177/02692163261416267

**Published:** 2026-02-25

**Authors:** Catriona R. Mayland, Ada Achinanya, Madeleine Harrison, Val Bryant, Sheila Payne, Linda Sharp, David Hamilton, Joanne M. Patterson

**Affiliations:** 1 University of Sheffield, UK; 2 Sheffield Teaching Hospitals NHS Foundation Trust, UK; 3 Palliative Care Unit, University of Liverpool, UK; 4 CHANGE PPI Group, Sunderland, UK; 5 Lancaster University, UK; 6 Newcastle University, UK; 7 Newcastle Hospitals NHS Foundation Trust, UK; 8 University of Liverpool, UK

**Keywords:** head and neck cancer, palliative care, qualitative research, delivery of healthcare

## Abstract

**Background:**

The disease trajectory for people with incurable head and neck cancer is unpredictable. This group also has high healthcare utilisation.

**Aim:**

To explore multi-perspectives views about incurable head and neck cancer healthcare experiences over time and how best to improve care.

**Design:**

A prospective, longitudinal qualitative study was conducted involving three head and neck cancer networks in Northern England. Maximum variation sampling of incurable head and neck cancer adult patients (⩾ 18 years) was conducted. Patients were invited to participate in up to three serial interviews (conducted every 4 months); caregivers could support interviews and were ‘proxies’ if the patient became too unwell or died. Online focus groups were conducted with healthcare professionals. Picker’s ‘Principles of Patient-Centred Care’ informed topic guides and framework analysis.

**Results:**

Forty-nine participants (18 patients, eight caregivers and 23 healthcare professionals) were involved in 44 interviews and 4 focus groups. Participants’ accounts revealed systemic variability in experiences of healthcare delivery especially in terms of medication access, caregiver preparedness and information needs. Navigating a ‘fragmented’ healthcare system was a key challenge, within both non-emergency and emergency situations and especially if living alone. Suggestions to improve healthcare experiences included improving clarity about key healthcare professional contacts and communication around prescribing, and diversifying means of healthcare team working.

**Conclusion:**

Issues relating to variability in experiences and challenges in healthcare system navigation, including access to symptom-relieving medication, impact on patient-centred care. Consideration to accessible means of seeking advice and support as well as improving caregivers’ preparedness are key priorities.

**What is already known about the topic?**

- The disease trajectory for people with incurable head and neck cancer is unpredictable.
- This cancer subgroup has high healthcare utilisation even in the last months of life.

**What this paper adds**

- Patients, caregivers and healthcare professionals consistently reported systemic variability in healthcare experiences for those with incurable head and neck cancer.
- Improving access to symptom relieving medications, helping more with advocacy, and developing ways to improve caregivers’ preparedness reflect areas for improvement across the disease trajectory.
- Information needs change over time, with initial overload, but then complexities relating to advance care planning developing later due to the unpredictable nature of the disease.

**Implications for practice, theory or policy**

- Solutions include cancer centres adopting more accessible, inclusive means of communication and providing patients and families with reliable contact points for key healthcare professionals.
- Developing strategies or interventions to improve caregivers’ preparedness should incorporate both caregivers and relevant healthcare professionals to ensure the technical aspects of care can be addressed.

## Introduction

Globally, head and neck cancer is the seventh most common cancer^1^ and due to factors, such as high frequencies of presentation with advanced disease and co-morbidities, 5-year survival remains at 50%–60%.^2^ Both the illness and treatment affect aspects of vital functioning such as breathing, speaking and swallowing.^3,4^ The disease trajectory is often complex and unpredictable.^5^

Those with incurable head and neck cancer experience multiple symptoms and have extensive care needs including pain, complex nutritional issues and airway management. They often have high healthcare resource utilisation including hospital or intensive care admission. Additionally, they have frequent ‘intensive’ interventions such as cardio-pulmonary resuscitation, chemotherapy, and tracheostomy placement.^6–9^ Compared with other cancer groups, people with incurable head and neck cancer are twice as likely to have multiple emergency department attendances in the last month of life.^10^

Overall, the evidence base for improving incurable head and neck cancer healthcare experiences is limited in quality and volume. Systematic reviews rarely focus on incurable disease and tend to link to the impact of an anticancer treatment.^11–13^ Two reviews, focussing on incurable head and neck cancer, identified that most studies were small scale and quantitative ^3,14^; the available qualitative studies were small (pooled total of 38 patients and 25 caregivers) and limited by cross-sectional design. No qualitative studies have explored healthcare services engagement nor captured experiences over time. Multi-perspective qualitative studies can offer a richer, nuanced understanding of a topic by considering diverse experiences and perceptions from different viewpoints.^15^ Hence, this study aimed to explore multi-perspectives views about healthcare experiences over time for those with incurable head and neck cancer and how best to improve care. Findings will inform co-design workshops to help develop potential solutions (to be published separately).^16^

## Materials and methods

### Design

A prospective qualitative study, involving longitudinal interviews and single time-point focus groups. A social constructivist approach was adopted viewing that knowledge and understanding is not inherent but built through our social interactions and cultural context.^17^ We used Framework Analysis, which is not aligned to a particular epistemology, and can incorporate an inductive methodology.^18^ The research was conducted by a multidisciplinary team with head and neck cancer clinical expertise (CRM, DH, JMP), ‘lived experience’ as a patient (VB) and qualitative healthcare service research (CRM, AA, MH, VB, SP, LS, DH, JMP).

### Setting

Within the UK, head and neck cancer care is managed across large networks in a ‘hub and spoke’ model. Three large hospitals, based in Sheffield, Liverpool and Newcastle in Northern England, represent central ‘hubs’ and were the chosen study sites. Multi-disciplinary meetings and anti-cancer treatments are undertaken in these ‘hubs’ for people living across a wide geographical area. The ‘hubs’ are supported by ‘spokes’ that is, outreach clinics and support teams providing care closer to home.

### Longitudinal interviews

#### Population

Patients were identified according to the following inclusion criteria: clinical diagnosis of incurable head and neck cancer; ⩾18 years old; reside within one of the three chosen cancer networks; and if receiving anti-cancer treatment, intent is palliative. Patients were excluded if they were unable to provide informed consent.

#### Sampling

Clinical colleagues conducted initial screening by reviewing their current out-patient clinic lists and in-patients against inclusion and exclusion criteria. Maximum variation sampling^19^ of patients was then undertaken using factors judged likely to influence experiences (e.g. distance from cancer centre, living circumstances). A matrix of these factors was constructed and used to target further recruitment.

#### Recruitment

Clinical colleagues initially mentioned the study during routine clinical reviews and shared preliminary study information and Participant Information Sheets. Two clinicians (CRM, DH) within the research team had clinical responsibilities for a small number of potential participants.

If verbal consent was given, local clinical research assistants (Sheffield, Liverpool) and clinicians (Newcastle, DH) approached potential participants during clinical reviews or via telephone.

If potential participants wished to consent in the study, then they could do so as soon as they perceived they had sufficient information. Hence, this allowed the option to consent on the same day they received the study information. Otherwise, research team members contacted potential participants within 2 weeks to further discuss the study.

Participants were asked to nominate a caregiver that is, family member/friend ⩾18 years old involved in their care and able to provide informed consent. This individual could either directly support the patient during their interview and/or be interviewed as a ‘patient proxy’ if they became too unwell to take part or died.^20^

Formal consent was obtained by the Research Associate (MH) prior to the first interview. During telephone calls to arrange subsequent interviews, participant’s willingness to continue was checked and then confirmed immediately before the actual interviews.

#### Data collection

Key demographic and clinical information were collected (gender, age, ethnicity, living circumstances, localised or metastatic disease, whether on anti-cancer therapy, postcode—to determine distance from cancer centre and deprivation status). Up to three serial interviews were conducted, approximately four-monthly, by trained qualitative researchers (MH, CRM). Whilst MH had no previous relationship with the participants, CRM, had previously known two of the participants but did not conduct their interviews. Depending on individual preference, interviews were face-to-face (at a place of the participants choice), online (including online written),^21^ or via telephone. Topic guides (Supplemental File 1) were informed by the eight aspects of Picker’s Principles of Patient-Centred Care (Textbox 1)^22^ and Patient and Public Involvement (PPI) input. This was obtained from our core research team member (VB), who has lived experience as a patient and family carer, as well as her PPI ‘CHANGE’ group. This ensured clarity of terminology and good flow for the interview. The guides focussed on healthcare experiences for both the patient and their caregiver and longitudinally, interviews asked about changes over time, challenges experienced and potential alternatives or solutions to these. For bereaved family members, the focus was on end-of-life care experiences.

**Textbox 1. Pickers principles of patient-centred care.**

● Fast access to reliable healthcare advice.

● Effective treatment by trusted professionals.

● Continuity of care and smooth transitions.

● Involvement and support for family and carers.

● Clear information, communication and support for self-care.

● Involvement in decisions and respect for preferences

● Emotional support, empathy and respect

● Attention to physical and environmental needs.

### Focus groups

#### Population

Any hospital, community or hospice-based healthcare professional (HCP) involved in the care of incurable head and neck cancer were invited to participate. This included doctors, nurses and allied health professionals for example, dieticians, speech and language therapists, who worked within specialised head and neck cancer or provided palliative care in any setting.

#### Sampling

Individuals were identified via email using existing collaborative network groups,^23^ local circulation lists, bulletins and social media. A purposive, snowball sampling strategy,^19^ based on role, speciality and care setting, was used to gain different perspectives across the three cancer networks.

#### Recruitment

Potential participants were sent emails enclosing the Participant Information Sheet and asked to contact the research team if there were interested in the study.

#### Data collection

All participants provided informed consent prior to the focus group being conducted. Key demographic data was collected (gender, age, ethnicity and role). Topic guides (Supplemental File 1), also informed by ‘Patient-Centred Care’,^22^ explored HCP views about perceived challenges for themselves, patients and caregivers as well as potential solutions. On-line focus groups were moderated by clinical academics (CRM, JMP) and supported by a trained researcher (AA).

All interviews and focus groups were digitally recorded and field notes were made immediately afterwards (MH, AA, CRM). Recordings were transcribed verbatim, and the transcripts anonymised. Findings are reported in keeping with the ‘Consolidated criteria for reporting qualitative research’ (COREQ).^24^

#### Analysis

Analysis was supported using NVivo (version 12) and conducted using a Framework approach^18^:

##### Familiarisation and initial coding

Initial familiarisation of transcripts/fieldnotes was undertaken (MH, CRM, AA) immediately after the interview. The wider research team (CRM, MH, JP, SP, VB) reviewed three transcripts, noting initial impressions. Open codes were assigned to relevant text.

##### Developing a working framework

The initial analytical framework was formed (MH), grouping conceptually related codes into categories, and linking these with Patient-Centred Care principles.^22^ The framework was subsequently piloted on four further transcripts (MH, CRM) followed by discussion and refinement of categories and codes.

##### Applying the framework

The refined framework was applied to all subsequent transcripts (MH, CRM, AA). Data was analysed for each individual interview at each time point and then reviewed as collective patient and/or family carer groups across each time point. Data from the single time-point focus groups was initially analysed separately and then compared and contrasted with all interview data. Focus group findings were used to corroborate interview findings or add new insight into specific areas.

##### Charting the data

All data was summarised and charted (AA) striking the balance between reducing data whilst retaining the original meaning and highlighting illustrative quotations. Charting was discussed with research members (CRM, JP, SP, VB) during team meetings.

##### Interpreting the data

The data summaries were reviewed (CRM, AA) to map connections between categories and explore relationships. Data synthesis was conducted by visually charting the data. Further mapping to show relationships between findings and ongoing discussions (AA, CRM, wider team) were used to refine themes.

### Ethics

Approval was granted by the Health Research Authority and the West Midlands-Solihull Research Ethics Committee (REF 23/WM/007), England (on 24.02.2023).

## Results

### Longitudinal interviews

Overall, 55 patients were screened and approached with a total of 26 individuals interviewed (18 patients and 8 family carers. Reasons for declining included: ‘too much going on’, ‘didn’t want to reflect on experiences’, ‘too distressing’, or didn’t respond to further contact. From 13 caregivers who provided consent, 8 were interviewed either jointly with the patient or as a patient ‘proxy’.

In total, 44 interviews were conducted between May 2023 and July 2024. This consisted of 18 first interviews, 14 second interviews (11 with patients +/– caregivers; three solely with caregivers); and 10 third interviews (nine with patients +/– caregivers; two solely with caregivers; Supplemental File 2). First interviews lasted between 19 and 76 min (mean 50.7 min) and follow-up interviews between 5-54 min (mean 27.0 min). During the study, six patients died, and one was deemed to no longer have incurable disease. Patient participants tended to be male and live in more deprived areas (Table 1). There was good diversity in terms of age, living circumstances, and the distance from their residence to the cancer centre. All except one self-identified as White British. Caregivers were mostly female and the patient participants’ spouse.

**Table 1.** Demographic and clinical characteristics of participants.

Characteristics	Longitudinal interviews		Focus groups
	Patients (*n* = 18)	Caregiver (*n* = 8)	Healthcare professional (*n* = 23)
Gender			
Female	2	7	21
Male	16	1	2
Age			
⩾65	10	2	1
<65	8	6	22
Self-identified ethnicity			
White British	17	7	18
White Irish/White other/Asian or Asian British/Any other Asian background^a^	1	1	5
Living circumstances			N/A
Lives alone	9	-	
With spouse/partner	9	8	
Relationship			N/A
Spouse	-	7	
Child	-	1	
Cancer stage^b^			N/A
Localised	8	N/A	
Metastatic	10		
Proximity to cancer centre			
Lives within city limits of cancer centre	10	N/A	N/A
Lives outside city limits of cancer centre	8		
Anti-cancer therapy			
On anti-cancer therapy	8	N/A	N/A
Not on anti-cancer therapy	10		
IMD^c^			N/A
>5	4	N/A	
⩽5	14		
Current area of work			
Doctors (representing oncology, surgery, palliative medicine, general practice)	N/A	N/A	8
Nurses (representing head & neck cancer, palliative care, community)			4
Allied health professionals/pharmacist			11

N/A: not asked or not applicable.

a Grouped together to protect identity.

b Types of cancer have not been specified to protect identity but included: laryngeal, oropharynx, tonsil, salivary duct, mandibular, hypopharynx, paranasal sinus/nasal cavity, metastatic SCC neck (occult primary), pharynx, lip/oral cavity/tongue and nasopharynx.

c IMD: index of multiple deprivation; range from 1 to 10 where decile 1 represents the most deprived 10% and decile 10 the least deprived 10%.

### Focus groups

Twenty-nine HCPs were approached and 23 participated in one of four focus groups which lasted between 63 and 78 min (mean 69.2 min). Participants were predominately female and aged <65 years (Table 1). There was diversity in terms of their ethnicity and role.

### Findings from the qualitative analysis

**Figure 1.**
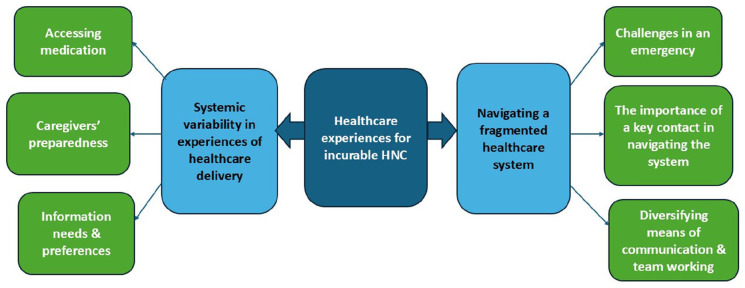
Healthcare experiences for those affected by incurable head and neck cancer.

Two main themes were developed. These were: ‘systemic variability in experiences of healthcare delivery’ and ‘navigating a fragmented healthcare system’; each had three subthemes (Figure 1).

### Systemic variability in experiences of healthcare delivery

Participants described three critical domains of variability: accessing medication, caregiver preparedness, and information needs and preferences.

#### Accessing medication

For some patients and caregivers, across the whole course of the illness, the process of obtaining medication was chaotic, unreliable and marked by substantial delays or shortage issues; this was especially the case for symptom-relieving medication.

‘So, *two* weeks last Tuesday, I ordered my prescription online, and it still hasn’t come. And my carer is coming at 1.30 pm to help me, but I’m having to ask them to ring and chase my medication. They rang up on Friday, and was told that it would be marked as urgent and I would get it that day. But not so, on Monday my carers rang the chemist, and I was told it would be Tuesday, **but it’s still not come**.’ (P15, first interview, lives alone)

At the end-of-life, when caregivers perceived their priority should be spending time with the patient, the challenges accessing medication prevented this.

‘. . .To get this oxycodone, and it was really stressing me out because he was in that much pain. . .I was just going round different chemists. . . . .It was ridiculous. I had to leave him practically every day **to go out hunting for this medicine**. . .’ (CO8, 2nd (after-death) interview)

This was congruent with HCPs’ views which echoed patients’ and caregivers’ perspectives about shortages and difficulty accessing specialised items. HCPs noted this could disrupt care, impact symptom management and create a stressful situation.

‘One of the things that I see a lot of is access to getting medicines and drug shortages and then that **causes undue stress for the patient and family** trying to find or get alternatives that may not be as suitable.’ (FG3, HCP4)

In contrast, a few patients, earlier in their illness, reported a well-coordinated system characterised by timely medication delivery and clear communication among healthcare providers and pharmacies.

‘Oh, that’s been, that’s been brilliant as well. If I’ve ever had to call them to give us extra medication because I’ve been going away or I’ve needed more, more of me (sic) medication . . . . . .there’s been no problem. . . . .**the chemist has been absolutely superb** as well and I think that is because the chemist is attached to the doctor’s surgery. . . . . .’ (P07, first interview, lives with spouse)

HCPs perceived that hospital teams providing primary care doctors with specific details for prescriptions for example, length of course of treatment, may alleviate any concerns about opioids and ensure patients received sufficient supplies.

‘. . . .the opioids. . . .just **specifying** that. . .this person’s medications has been titrated, and this is the level that what we, we expect it to be that for a period of time **can you please prescribe them a month’s worth of this at a time**. . . . .just writing that you would want them to do that. . .’(FG3, HCP4)

#### Caregivers’ preparedness

Patients, family caregivers and healthcare professionals described the ‘*burden*’ caregivers experienced both physically and emotionally when supporting someone with head and neck cancer and the significant changes this meant for day-to-day life. Reports were given of being overwhelmed and insufficiently prepared to manage complex needs. The emotional and practical burdens were substantial, particularly when caregivers lacked comprehensive training or support. Practical challenges including technical tasks like suctioning, managing tracheostomies and feeding tubes, added layers of complexity and responsibility.

‘. . .he basically just come home, brought him home, and with his trachy in. . . .they trained me up to do that in hospital. . . .it was just too much, you know, with suctioning, I had a suctioning machine, all the pipes, everything. It was just, it was just, **it was really full-on care**.’ (C08, 2nd (after-death) interview)

These challenges could extend into care at the very end-of-life and were amplified if there were acute changes in the patient’s condition. Other caregivers, reflecting on their end-of-life experiences, illustrated different scenarios where there was a more progressive and predictable decline and additional community HCP support was available. Hence, the caregivers were able to participate effectively in care and felt equipped with the knowledge of what to do if their situation changed.

‘. . . .as [PATIENTS NAME] were getting weaker, you know, [DISTRICT NURSES & PALLIATIVE CARE NURSES] had discussions with me. . . that **exactly what I’d need to do when that time come**. And he had a care plan in place where it explained it, so you knew. . . .’ (C02, 3rd (after-death) interview)

#### Information needs and preferences

The way information was shared by HCPs including the format, timing and content shaped patients’ and caregivers’ experiences. Patients, family caregivers and healthcare professionals described the vast volume of information that ‘*all gets thrown at them*’ initially following diagnosis, about treatment and need for interventions for example, feeding tube insertion. For patient participants especially, there was a lack of alignment between information delivery and individual preferences, with information not always tailored to their needs—being perceived as insufficient, ill-timed or overwhelming. Examples included discussions about treatment side effects and unsolicited prognostic information, which caused emotional distress.

‘Well, they give me this pack here and I started to read through it, a little each day, to find out about it and after *three* weeks. . . . . .**I was too terrified of what side effects.** I had to phone me (sic) [SPECIALIST NURSE] and she reassured us. . . .and that helped a lot.’ (P06, first interview, lives alone)

In contrast, other patient participants expressed that they experienced concise, well-timed information aligning with their immediate needs, and shared their experiences of clear explanations and treatment options.

‘I don’t think you can play with half a deck.. . . . . . .**I would rather have the information**, and then I just view it as a problem that just needs sorting. And you can’t really sort any problems out until you know what the problem is, so.’ (P12, first interview, lives alone)

Specific complexities that were described later in the illness, related to the unpredictable ‘*pace of change*’ which could impact on advance care planning; the volume of different issues to address for example, management of feeding; the number of different healthcare professionals involved in care; and the need to review and revise pre-existing communication strategies.

‘. . .that might be people who have had strokes previously and then the progression of disease means that those symptoms often, their communication deteriorates on top of that, so strategies that were in place don’t work so well. . . . . . . .how quickly things can change and how many things there are to communicate about your journey as you go along. . . . . .and family members may have different ideas about what they think is the best course of action.’ (FG1, HCP5)

### Navigating a fragmented healthcare system

Fragmented care was described by patients, caregivers and HCPs as a central challenge when navigating healthcare systems. This encompassed discontinuity in care, lack of coordinated support, and variable patient-provider interactions. Those who lived alone or had a limited social network especially felt the impact. Three sub-themes were developed: challenges in an emergency, the importance of a key contact in navigating the system, and diversifying means of communication and multi-disciplinary team working.

#### Challenges in an emergency

Specific challenges occurred during times of crisis at home. Some patients and caregivers reported their default was to call an ambulance or attend the emergency hospital department. This stemmed from a lack of confidence or knowledge of alternative options.

‘. . . .so I think **my main point of contact to be honest is 999** [emergency number] you know what I mean, which would be logical speaking wouldn’t it like. And so, that’s why I said if I got nurses coming a bit more often they’d be able to see how I was.’ (P04, first interview, lives alone)

This view was echoed by HCPs who reported that, despite there being alternative community services, patients may default to emergency department attendance.

‘I still find patients, you know, especially, like, PEG problems [percutaneous endoscopic gastrostomy], back and forth. And then **they end up going to A&E** [emergency department] when there is (sic) services out there that can help them.’ (FG2, HCP3)

One important reason related to the frightening nature of emergencies that could arise such as acute breathing difficulties for example, airway obstruction, and bleeding.

‘. . . . . .**he started having breathing difficulties** and I took him to hospital and that’s when they ended up putting a tracheostomy in. . . . . . .they told us it were palliative and everything. . . . .just obviously he’d choke to death without it. It was really traumatic. . . . .’ (C08, 2nd (after-death) interview)

Even in the context of proactive advance care planning which may occur later into the disease trajectory, in an emergency situation, hospital was perceived as a place of safety by patients, caregivers and HCPs, meaning admission was unavoidable.

‘. . . . .we try to do our respiratory clinical management plan as early as possible. . . . .**when an emergency actually happens it’s frightening**. . .trying to actually follow their wish at the time that they didn’t want to come to the hospital, but actually airway obstruction is so scary that they end up coming to the hospital, it’s very difficult to stop them doing that.’ (FG1, HCP2)

#### The importance of a key contact in navigating the system

Patient and caregivers reported being uncertain about pathways for accessing non-emergency care, a view re-iterated by HCPs who perceived they were *‘yo-yoing back and forth amongst services’ (FG3, HCP3).*

‘. . .if I would have picked the phone up today and asked, what can I do next? Can you point me in the right direction? **I wouldn’t know who to contact**. . . .I really wouldn’t know my first point of contact.’ (P09, first interview, lives alone)

Patients, caregivers and HCPs reported the need for a ‘navigator’ or key contact to signpost and support individuals to find their way around the system and help access information and support.

‘I think **having that central point of contact**, and maybe some training for that person around kind of the routes which they would need to access for people to get certain support for acute and community.’ (FG2, HCP4)

Generally, the key contact (when provided) was an HCP, often the specialist HNC nurse, or a network of different professionals. For some participants, a caregiver was their key navigator, advocating on their behalf. As the illness progressed, having consistent contact points provided security and reduced feelings of uncertainty and helplessness.

‘Well, I think because I know the signs now and I know because the nurses are 24/7, we can ring them anytime…**I’ve got that big safety net**, so if I’ve got the feelings somethings not right compared to normal, I ring them and I’ll explain and. . .they’ll come out, or they’ve sent a doctor out, who comes, they come out so quickly.’ (C07, third interview, patient & caregiver)

#### Diversifying means of communication and multidisciplinary working

As well as the need for a key ‘advocate’ or ‘coordinator’, ideas for potential solutions to aid navigation and care coordination included expanding the role of information technology and digital tools, whilst appreciating variability in digital literacy. Patient participants reported that General Practitioner ‘*alerts*’ as to who was approaching end-of-life and connected electronic record systems would be beneficial.

‘I want to see and you want to see, everyone to see the same file, with every information’ (P19, first interview, patient and carer)

Other ideas from HCP included utilising existing Apps and electronic alert systems to aid communication between healthcare teams for example, for prescriptions, or to inform other teams a patient had been admitted to hospital. Consideration was given to more accessible ways to aid patient-to-HCP communication like using ‘WhatsApp’ and visual methods of providing information for example, ‘YouTube’ videos.

‘Well, everyone watches YouTube don’t they, you know, YouTube video? I don’t know, even I know that there are some cohorts that really don’t have that good access to internet and smartphones and things like that, but yeah, I don’t know. **Watching things, everyone, you know, it’s very powerful isn’t it so?’** (FG1, HCP6)

Considering more innovative ways of working to help ‘bridge the gaps’ between different care settings was discussed. As well as cross-boundary working, utilising hybrid methods of reviews for example, combining face-to-face and online consultations involving generalist and specialist HCPs, was thought to be beneficial. One example provided was where a healthcare worker with general skills was visiting HNC patients at home and supported by a more highly trained speech and language therapist via a remote video call.

‘we have a full-time assistant practitioner funded just for head and neck cancer who works across speech therapy and dietetics. **And so, she has more time and flexibility to go out to a patient’s home, and we video call her.** And that means that that just takes up *one* of my outpatient slots while she travels around. (FG1, HCP3)

## Discussion

### Main findings

Improving access to symptom relieving medication, providing better support for caregivers and helping more with advocacy reflect areas for improvement across the incurable head and neck cancer disease trajectory. Information needs change over time but are notable in terms of the initial volume and complexity and the later challenges relating to the unpredictable pace of change in the disease. Having more accessible means of communication and key points of contact with HCP could provide practical help, especially if there are speech difficulties or limited support networks. Whilst advance care planning is essential, particularly for those at risk of breathing or bleeding difficulties, hospitals can be the most appropriate and safe place of care.

### What this study adds

Access to pain relief medication is recognised as a huge inequity in global health, where low-income to middle-income countries have limited access to oral morphine.^25^ This study demonstrates that within a Western European country, with a publicly funded healthcare system where prescribed medications are free of charge for people with cancer, there are also issues accessing critical medication. This impacts on the adequacy of symptom control, including at the end-of-life, and causes patient and caregiver distress. Suggestions for improvement such as shared access to electronic prescribing systems, team integration and enhanced pharmacy provision resonates with findings from previous research.^26,27^ Our study also suggests that enhancing communication, especially between cancer centres and community HCPs, is needed to streamline opioid prescribing.

Enhancing the support given to prepare caregivers was a key area identified for improvement. Context is important as although individuals may be comfortable assisting with suctioning in a hospital environment, this can be perceived very differently when at home with limited immediate support. This resonates with findings from previous studies within palliative care and advanced cancer.^28,29^ One head and neck cancer study reported caregivers were least comfortable helping with ‘medical aspects’ of care such as feeding and breathing tubes.^30^ Optimising training for community HCPs around technical aspects of care is needed to provide help when caregivers seek advice. Transferable learning from interventions which helped improve transitions in care from hospital may be beneficial.^31^

Whereas identifying patients’ priorities has previously occurred using ‘concerns inventories’^32^ or electronically monitoring symptoms,^33^ there is a need to identify caregiver support and information needs. Potential mechanisms to address this include the routine use of assessment tools specifically for caregivers^34^ and more comprehensive training and support. Training should include the caregiver as well as the HCP who will support them, especially within the community setting. Developing and evaluating interventions in these areas reflect priorities for future research.

Social isolation and low health literacy (i.e. a person’s ability to ‘obtain and translate knowledge and information in order to maintain and improve health’)^35^ are associated with higher mortality.^36^ Our patient participants who lived alone or had limited social networks perceived issues of inaccessibility. Furthermore, where there are challenges in physical abilities to communicate, these issues can be perceived more acutely.^37^ By acknowledging key areas of variability in patient experiences, the factors driving these inequities can be considered; this, in turn, may help determine the optimum ways in which improvements can be made.

### Strengths and limitations

This is the most comprehensive, qualitative study assessing multi-perspective incurable head and neck cancer healthcare experiences and, to our knowledge, the only one to use a longitudinal approach.^38,39^ It has broadened understanding on important unmet needs (e.g. challenges in accessing medications), which occurred across the disease trajectory, and which have only started to receive more attention.^26,40^ Additionally, the study’s findings resonate with the recent Lancet Oncology commission report—the fragmented and impersonal systems and the absence of connection and relational care.^41^ While there are technological and treatment advances, these should not be sacrificed at the expense of ensuring equitable access to palliative care, aiding navigation and supporting changing information needs.

We recruited patients and caregivers who were diverse in age, socioeconomic deprivation, living circumstances, and the distance of their residence from the cancer centre. Most participants were men reflecting the wider HNC population.^42^ The longitudinal nature enabled issues identified earlier in the disease trajectory to be followed through. Specific head and neck cancer challenges demonstrating different complexities over the course of the illness include the provision of information—there is an initial overload of information; but later limitations to satisfactory advance care planning can arise due to the unpredictable disease trajectory, the need to revise communication strategies and the vast number of different individual, clinical and social factors to consider when making plans. By building trusting relationships, patients reflected on their experiences and shared what might have improved these for example, ‘an advocate’ to help navigate care. Encapsulating bereaved relatives views^43^ provided unique insights into care surrounding death, which otherwise would not have been possible.

We included a range of methods for data collection to ensure accessible, inclusive options. As well as more traditional methods, online written interviews were offered^21^ and modified approaches used for example, enabling texting (then reading aloud messages for transcription), writing and signing, and pacing the interview into short segments.

Recruitment had challenges. We were keen to approach individuals as soon as they were given an incurable head and neck cancer diagnosis. Sensitivity and judgement had to be considered, however, as it was challenging to discuss research participation at this timepoint. Gatekeeping may have influenced which patients were approached^44^ and generally recruitment was better when approached by clinician who had a prior relationship (while emphasising there was no obligation). Only one participant did not identify as being ‘White British’ despite additional efforts to recruit from ethnically diverse communities. As individuals from ethnically diverse communities with cancer are known to have worse healthcare experiences,^45^ this limits the diversity of cultural experiences, and the understanding of engagement and use of services.

The study was undertaken within Northern England, in areas with high head and neck cancer incidence. Findings are likely to have most relevance internationally for societies with nationally funded healthcare systems, although issues like access to key medications have global application.

## Conclusions

Our findings highlight the systemic variability in healthcare service experiences of people with incurable head and neck cancer and the challenges in system navigation and access to medication. There is a need to provide connection and relational care which helps address changing informational needs. Providing accessible means of contacting healthcare teams may be an initial step to help overcome challenges. Improving family members’ preparedness for caregiving is a priority, and a focus on the potential ways to drive this is required to address these challenges and variability in care.

## Supplemental Material

sj-docx-1-pmj-10.1177_02692163261416267 – Supplemental material for Multi-perspective views about healthcare experiences for those with incurable head and neck cancer: A prospective, longitudinal, qualitative study Supplemental material, sj-docx-1-pmj-10.1177_02692163261416267 for Multi-perspective views about healthcare experiences for those with incurable head and neck cancer: A prospective, longitudinal, qualitative study by Catriona R. Mayland, Ada Achinanya, Madeleine Harrison, Val Bryant, Sheila Payne, Linda Sharp, David Hamilton and Joanne M. Patterson in Palliative Medicine

sj-docx-2-pmj-10.1177_02692163261416267 – Supplemental material for Multi-perspective views about healthcare experiences for those with incurable head and neck cancer: A prospective, longitudinal, qualitative study Supplemental material, sj-docx-2-pmj-10.1177_02692163261416267 for Multi-perspective views about healthcare experiences for those with incurable head and neck cancer: A prospective, longitudinal, qualitative study by Catriona R. Mayland, Ada Achinanya, Madeleine Harrison, Val Bryant, Sheila Payne, Linda Sharp, David Hamilton and Joanne M. Patterson in Palliative Medicine
