# Supplementary material for: Multi-perspective views about healthcare experiences for those with incurable head and neck cancer: A prospective, longitudinal, qualitative study
Source: Palliat Med. 2026 Feb 25;40(4):524–34. doi: 10.1177/02692163261416267 (PMC13062449; doi:10.1177/02692163261416267)
Supplement: sj-docx-1-pmj-10.1177_02692163261416267 – Supplemental material for Multi-perspective views about healthcare experiences for those with incurable head and neck cancer: A prospective, longitudinal, qualitative study [file sj-docx-1-pmj-10.1177_02692163261416267.docx]

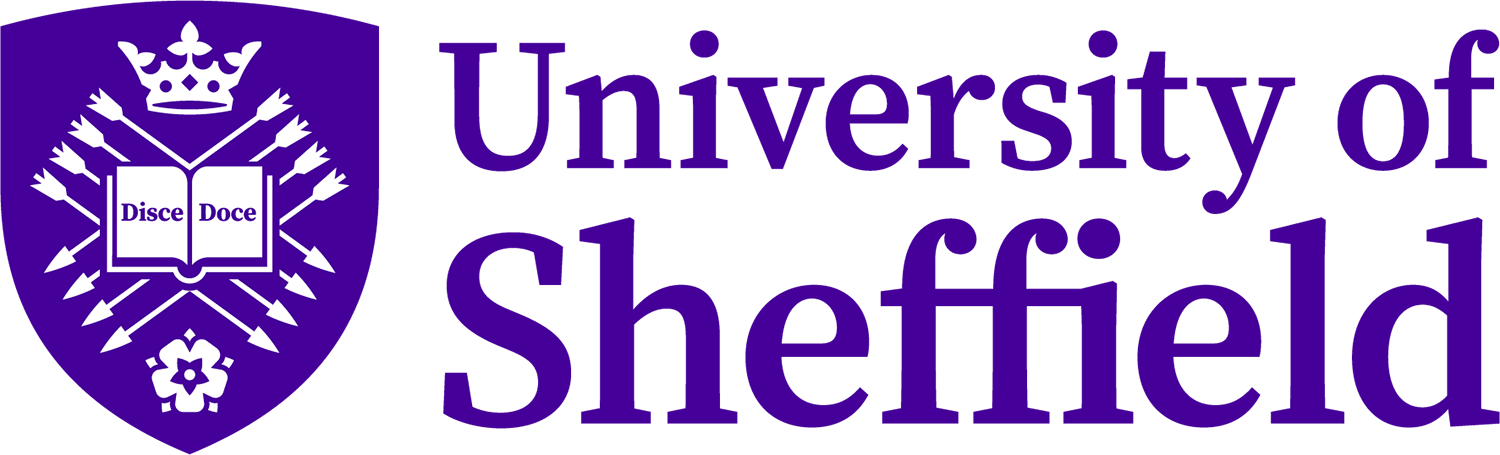


**Supplementary file 1. Topic guides**

**Improving incurable head and neck cancer healthcare experiences study**

**Patient topic guide: first interview**

**Introduction**

- Introduce researcher and research study (talk through key points from participant information sheet)
- Opportunity for questions
- If not already completed online, obtain written (or verbal for telephone/online interview) informed consent
- If not already completed online, ask patient to complete demographic information form
- Let participant know that when we ask questions about the clinical team this is a catch all term for doctors, nurses, therapists or anyone else currently providing care

**Interview questions**

Understanding patients’ needs

- Can you tell me where you were when you found out your cancer was incurable?
- How well do you think your symptoms have been managed since you found out your cancer was incurable?
  - Have you got any unmet physical care needs that haven’t been addressed by your clinical team?
- What emotional support have you needed since you found out your cancer was incurable?
  - Who has provided emotional support?
  - Do you have any unmet emotional/psychological needs?

Understanding patients’ healthcare experiences

- Can you tell me about the care you are receiving from your clinical team at the moment?
- Who has been involved in providing your care? Prompt: key point of contact?
- Have you mainly seen the same or different members of the clinical team?
  - Same: can you describe the relationships you’ve built?
  - Different: how has that worked? Do you think members of the clinical team have successfully shared information about your care?
- Have you moved between teams/ had hospital admissions?
  - How have transitions between teams/ in-patient and out-patient settings been handled?
- How easy do you find it to access the treatment and support you need?
  - Prompts: routine appointments/ crises points
  - Can you tell me about a time when you needed to seek some specific advice?
  - At times when you have become more unwell, who have you contacted?
  - Could you describe how your clinical team have discussed your treatment and care with you?
- When you were told the HNC was incurable, how did the member of the clinical team who spoke with you provide you with information? (prompts: verbal/ written/ volunteered/ ask questions/ information understandable?)
- Have your clinical team helped to ease your fears and anxieties? How?
- To what extent do you have confidence in your clinical team? Why?
- At any point were you asked by any member of your clinical team about your needs or concerns?
  - How did they respond to your needs/concerns?
- Did you have the opportunity to share your preferences with your clinical team? Prompts: background, social, cultural, religious values
  - How did the member of your clinical team respect your preferences?
- Were there particular issues with the environments in which you received care (Prompts: in-patient hospital care / clinic / home; issues with comfort / privacy)
- Is there anything that could have been done differently to improve your healthcare experience?

Understanding carers’ experiences

- To what extent have your family and friends been involved in your treatment/care?
  - How did staff encourage their involvement?
  - What have been the barriers to family/friend involvement?
- Have family/ friends been offered any support?
- If joint interview including a carer, ask the carer if there is anything they would like to add from a carer perspective?

**Debrief**

- Thank for participation
- Remind that we will be in touch in 3 months to arrange the next interview


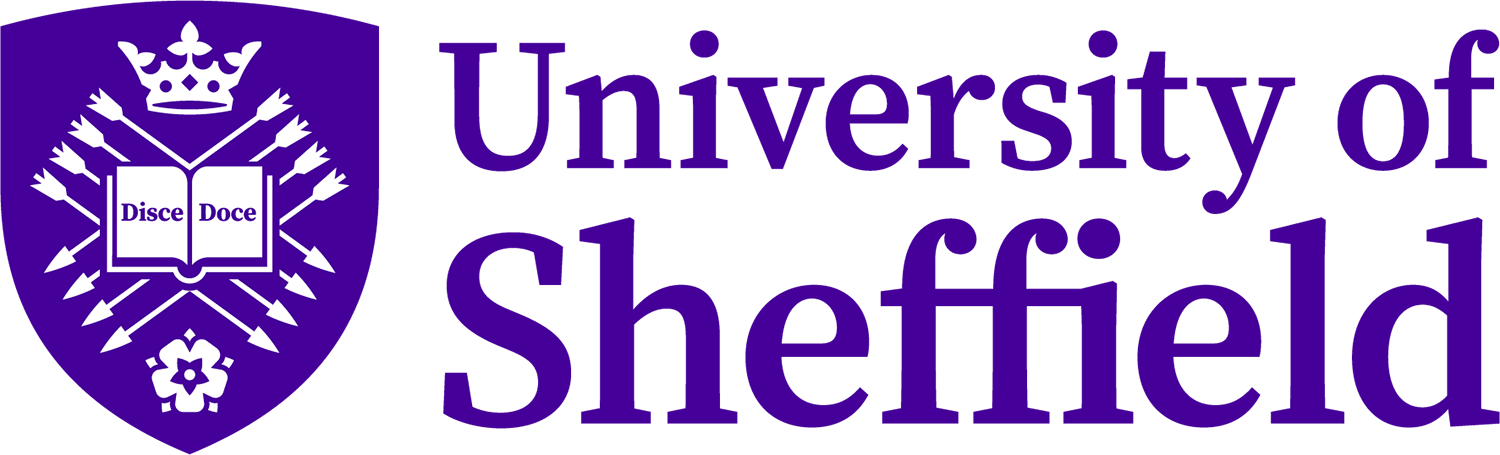


**Improving incurable head and neck cancer healthcare experiences study**

**Patient topic guide: second/third interview**

***Introduction***

- Re-introduce self and research study
- Opportunity for questions
- Obtain verbal consent for participation in this interview

**Changes since the last interview**

- How have you been since we last spoke?
- Have your physical needs changed at all since we last spoke?
- Emotionally, have there been any changes since we last spoke?
- Have you had much contact with your clinical team since we last spoke?
  - Prompts: clinic/ inpatient/ community/ GP
  - Prompts: how easy was access/ routine appointments or admissions/ crisis or emergency admissions/ same or different medical team/ friends or family involved
  - Can you think of any recent interactions with your clinical team that have stood out as being particularly good/bad? Why?
- Have you experienced any challenges accessing the care you have needed at the right time?
- Can you think of any way this could have been avoided? Or could be improved for others?
- If carer joint interview, is there anything you would like to add from your perspective?

**Debrief**

- Thank for participation
- If second interview, remind that we will be in touch in 3 months to arrange the next interview
- If third interview, remind them that we might be in touch about the workshops and we will send out a summary of results once the study is complete

[INSERT TRUST LETTERHEAD]
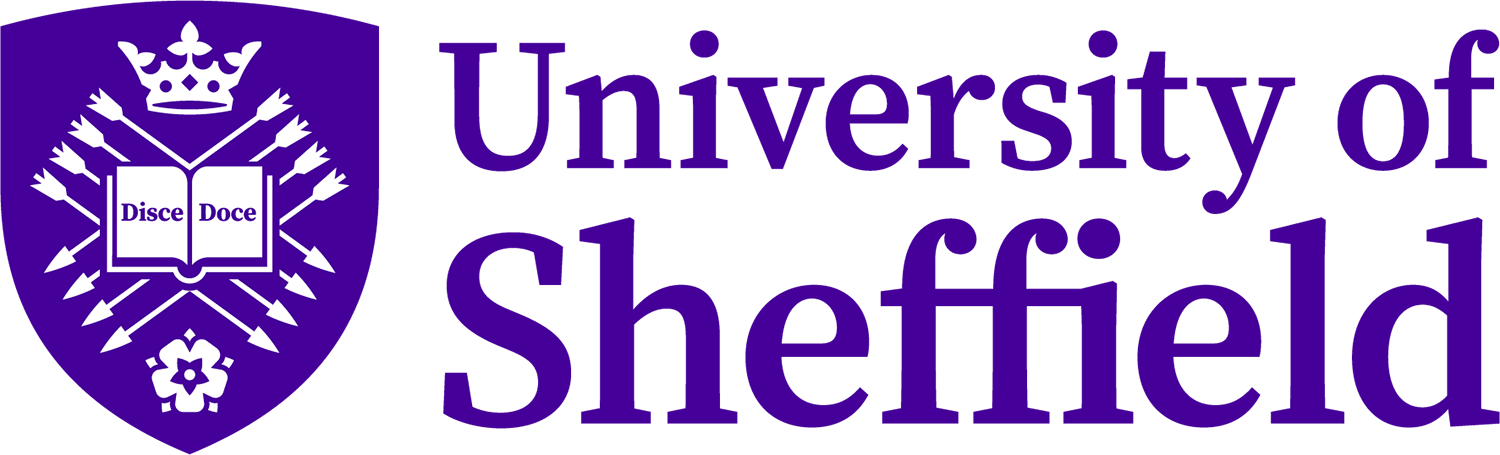


**Improving incurable head and neck cancer healthcare experiences study**

**Carer-only topic guide: second/third interview**

**Interview questions for bereaved family carers**

**Introduction**

- Express condolences
- Discuss that this might be a difficult conversation to have and reiterate that we can stop at any time
- Re-introduce self and research study
- Opportunity for questions
- Obtain verbal consent for carer participation in this interview

**Interview questions about the period since the last interview**

- Can you tell me about how X’s symptoms or general condition changed after we last spoke?
- Can you tell me about the care X received between our last interview and when they died?
- Were there any problems or challenges accessing the care X needed at the right time?
- Can you think of any way these challenges could have been avoided? Or could be improved for others in a similar situation?
- Did you have the information you needed and discussions about what to expect as X became more unwell?
- Were you involved in making decisions about what was important to X and you?
- Did you have any input from the specialist palliative care team (e.g. Macmillan/ Hospice/ etc.)? Or was care provided by the GP / District Nurse? Or X’s regular clinical team?
  - Can you tell me a little bit about the care they provided?
  - Did the different teams work together?
- Do you feel you have been supported throughout this period?
- At the very end-of-life, was X in the place of care they wanted to be?
- Were there any other challenges you or X faced during this time? Can you think of any way these challenges could have been avoided? Or could be improved for others in a similar situation?

**Debrief**

- Thank for participation
- Let them know that we might be in touch about the workshops and we will send out a summary of results once the study is complete


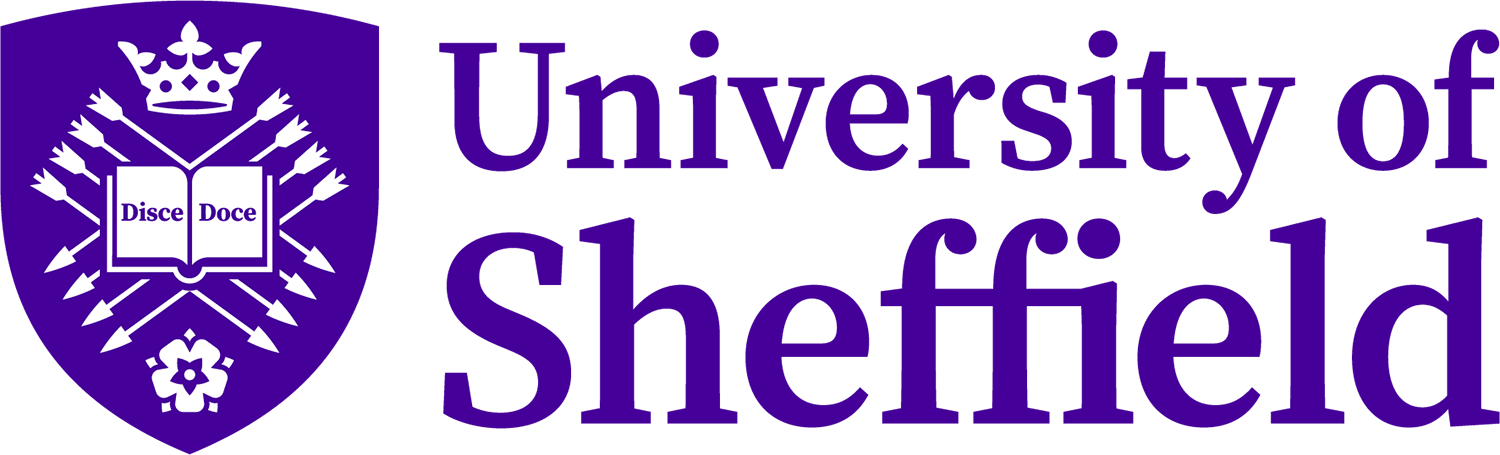


**Improving incurable head and neck cancer healthcare experiences study**

**HCP focus group topic guide**

**Introduction**

- Introduce researcher and observer, and describe their roles, introduce research study (talk through key points from participant information sheet) and other focus group members
- Stress confidentiality of discussion (Chatham House rule)
- Opportunity for questions
- If not already completed online, obtain written (or verbal for telephone/online interview) informed consent
- If not already completed online, ask participant to complete demographic information form

**Conduct ice-breaker**

Ask people to say their first name and their favourite place in the UK.

**Introduce format**

We are going to use principles of patient-centred care to help guide us through this focus group. We are keen to hear about any challenges you perceive and any potential alternatives or solutions that would help provide better patient-centred care for those with incurable head and neck cancer.

**Interview questions**

- Thinking about a patient with incurable head and neck cancer you have known, what were their main challenges with healthcare?

**Healthcare service provision**

- What do you see as potential challenges for incurable HNC patients when trying to access advice about their healthcare needs?
  - Prompt: routine care; unplanned crisis; timeliness of advice; location of hospitals/clinics; *(staffing)*
  - Prompt: what issues (if any) do you see in how the current pathways of care operate?
- What alternatives or solutions might be possible?
- Can you describe how providing continuity of care may be challenging?
  - Prompts: Where does this tend to occur (within/between different care settings; after hospital discharge; other situations)
- What about providing continuity in terms of information (provision and receipt)?
  - Prompts: When is this challenging (between different healthcare professionals / specialties; across different healthcare settings)?
- What alternatives or solutions might be possible?

**Communication and decision making**

- What challenges do you face when providing information to patients with incurable HNC?
  - Prompts: Timeliness; level of information to provide; best way to provide it
  - Prompts: Particular areas of information (clinical status; prognosis; processes of care; promoting self-care)
- What alternatives or solutions might be possible?
- What challenges do you face when involving incurable HNC patients in decision-making?
  - Prompts: Complexities that arise due to social / cultural values; literacy and understanding; other factors
- What might help improve patient involvement in decision-making?

**Meeting the needs of patients and family members**

- In terms of establishing good relationships with incurable HNC patients, can you share any particular challenges you experience?
  - Prompt: factors which influence relationships; social/cultural factors; *(training)*
- What might help improve relationships?
- Can you share any particular challenges you’ve experienced when supporting a patient’s physical needs?
  - Prompts: e.g., pain; managing other symptoms; activities of daily living
- Are there any challenges about the environments in which care is being provided?
  - Prompts: Which care setting? Are the issues related to comfort; privacy; dignity?
- What alternatives or solutions might be possible?
- Can you share any particular challenges you’ve experienced when supporting a patient’s emotional needs?
  - Prompts: e.g., anxieties about illness; treatment and prognosis; financial concerns
- What might help improve the provision of emotional support?
- Can you share any challenges in supporting and involving family members and carers in the overall provision of care?
  - Prompts: involvement in decision-making; supporting their own needs
- What might help improve the support of family members and carers?

Are there any other alternatives or solutions that haven’t been mentioned which would help improve healthcare experiences for incurable HNC patients?

**Debrief**

- Thank for participation
- Provide information about the next stage of the research
