# Supplementary material for: Multi-perspective views about healthcare experiences for those with incurable head and neck cancer: A prospective, longitudinal, qualitative study
Source: Palliat Med. 2026 Feb 25;40(4):524–34. doi: 10.1177/02692163261416267 (PMC13062449; doi:10.1177/02692163261416267)
Supplement: sj-docx-2-pmj-10.1177_02692163261416267 – Supplemental material for Multi-perspective views about healthcare experiences for those with incurable head and neck cancer: A prospective, longitudinal, qualitative study [file sj-docx-2-pmj-10.1177_02692163261416267.docx]

**Supplementary File 2. Overview of interviews conducted with patient and/or family carers over study recruitment period**

| Year | 2023 | | | | | | | | 2024 | | | | | | | | | |
| --- | --- | --- | --- | --- | --- | --- | --- | --- | --- | --- | --- | --- | --- | --- | --- | --- | --- | --- |
|  | 1 | 2 | 3 | 4 | 5 | 6 | 7 | 8 | 9 | 10 | 11 | 12 | 13 | 14 | 15 | 16 | 17 | 18 |
|  | May | June | Jul | Aug | Sept | Oct | Nov | Dec | Jan | Feb | Mar | Apr | May | June | Jul | Aug | Sept | Oct |
| P01 | Gave consent - but then declined interview | | | | | | | | | | | | | | | | | |
| P02 | I1 - P&C |  |  |  | I2 - P&C |  | Patient RIP |  |  | I3 - C only |  |  |  |  |  |  |  |  |
| P03 |  | I1 - P&C |  |  |  | I2 - P&C |  |  |  | I3 - P&C |  |  |  |  |  |  |  |  |
| P04 |  | I1 - P only |  | Patient RIP |  | Unable to contact family carer for post-death interview | | | | | | | | | | | | |
| P05 |  | I1 - P&C |  |  |  |  | I2 - P&C |  |  | I3 - P&C |  |  |  |  |  |  |  |  |
| P06 |  | I1 - P only |  |  |  |  | I2 - P only |  |  | I3 - P only |  |  |  |  |  |  |  |  |
| P07 |  |  | I1 - P&C |  |  |  | I2 - C (P unwell) |  |  |  | I3 - P&C |  |  |  |  |  |  |  |
| P08 |  |  | I1 - P&C |  |  |  | Patient RIP |  |  |  | I2 - C only |  |  |  |  |  |  |  |
| P09 |  |  |  |  | I1 - P only |  |  |  | I2 - P only | Patient no longer deemed to have incurable disease, so no further interview conducted | | | | | | | | |
| P10 |  |  |  | I1 - P only |  |  |  |  | Patient RIP | No family carer gave consent for interview | | | | | | | | |
| P11 |  |  |  |  | I1 - P only |  |  |  | I2 - P only |  |  |  | I3 - P only |  |  |  |  |  |
| P12 |  |  |  |  | I1 - P only |  |  |  | I2 - P only |  |  |  | I3 - P only |  |  |  |  |  |
| P13 |  |  |  |  | I1 - P only |  |  |  | I2 - P only |  |  |  | I3 - P only |  |  |  |  |  |
| P14 |  |  |  |  |  | I1 - P&C |  | Patient RIP |  |  | I2 - C only |  |  |  |  |  |  |  |
| P15 |  |  |  |  |  | I1 - P only |  |  |  |  | Patient RIP | No family carer gave consent for interview | | | | | | |
| P16 |  |  |  |  |  | I1 - P only |  |  |  | I2 - P only |  |  |  | I3 - P only |  |  |  |  |
| P17 |  |  |  |  |  |  | I1 - P only |  |  |  | I2 - P only |  |  |  | I3 - P only |  |  |  |
| P18 |  |  |  |  |  |  | I1 - P&C |  |  |  |  | I2 - P only |  |  | I3 - C only (P unwell) |  |  |  |
| P19 |  |  |  |  |  |  |  |  |  | I1 - P&C |  |  |  | Unable to find suitable time for 2^nd^ patient interview due to having anti-cancer treatment | | | | |

I = interview; P = patient; C = Family Carer
